# A Multistep, Multicomponent Extraction and Separation Microfluidic Route to Recycle Water-Miscible Ionic Liquid Solvents

**DOI:** 10.1021/acs.iecr.3c03312

**Published:** 2023-12-20

**Authors:** Bin Pan, Lanja R. Karadaghi, Richard L. Brutchey, Noah Malmstadt

**Affiliations:** † Mork Family Department of Chemical Engineering and Materials Science, University of Southern California, 925 Bloom Walk, Los Angeles, California 90089-1211, United States; ‡ Department of Chemistry, University of Southern California, 840 Downey Way, Los Angeles, California 90089-0744, United States; § Department of Biomedical Engineering, University of Southern California, 1042 Downey Way, Los Angeles, California 90089-0260, United States; ∥ USC Norris Comprehensive Cancer Center, University of Southern California, 1441 Eastlake Ave, Los Angeles, California 90033, United States

## Abstract

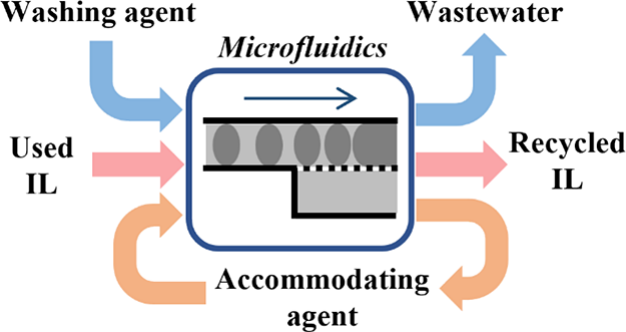

Recycling ionic liquid (IL) solvents can reduce the lifecycle cost of these expensive solvents. Liquid–liquid extraction is the most straightforward approach to purify IL solvents and is typically performed with an immiscible washing agent (e.g., water). Herein, we describe a recycling route for water-miscible ILs in which direct recycling is usually challenging. We use hydrophobic ILs as accommodating agents to draw the water-miscible IL from the aqueous washing stream. A biphasic slug flow of the mixed ILs and water is then separated by using a membrane. The water-miscible IL can then be drawn out from the mixed IL phase with acidified water and dried under vacuum. Both the water-miscible IL and the accommodating agent are then recycled. Here, we demonstrated a proof-of-concept of this process by recycling 1-butyl-3-methylimidazolium trifluoromethanesulfonate (BMIM-OTf) in the presence of the accommodating agent 1-butyl-3-methylimidazolium bis(trifluoromethylsulfonyl)imide (BMIM-NTf_2_) and acidified water. We then demonstrated the capacity to recycle 1-butyl-1-methylpyrrolidinium triflate (BMPYRR-OTf) from a realistic synthetic application: Pt nanoparticle synthesis in the water-miscible IL.

## Introduction

Organic solvents are key to chemical engineering practices, including organic-phase reactions, due to the solvation effect that increases reaction rates.^1^ Solvents can also be found extensively in processes of extraction and purification, where they strongly interact with the target compounds through intermolecular forces.^2^ Among organic solvents, volatile organic compounds (VOCs) are broadly used because of their wide availability and low cost; however, VOCs, such as benzene, exert long-term detrimental effects on the environment due to their high vapor pressures and low water solubility, while the emissions have to be consistently monitored and are regulated by authorities worldwide.^3−5^ Researchers have been studying sustainable solvents to replace these traditional VOCs; these efforts include, but are not limited to, exploring biomass-based renewable solvents,^6^ deep eutectic solvents (DES),^7^ and ionic liquids (ILs).^8^ ILs are molten salts, generally liquid at room temperature, composed of customizable combinations of organic cations and organic or inorganic anions.^9^ ILs are regarded as candidates for green solvent replacements of VOCs due to their extremely low vapor pressures and emissions to the atmosphere,^10^ as well as great chemical and thermal stability (often to temperatures of >300 °C).^11^ Despite the merits of ILs as non-VOC solvents, their expanded applications are limited by their high cost^12^ and, surprisingly, potential environmental impacts.^13^ For example, Clarke and co-workers reported that the superior stability of ILs can make them persistent environmental pollutants.^14^ Recycling paves a new path to reduce both the lifecycle costs and the downstream discharge of used ILs, and a variety of efforts have been undertaken to recycle used IL solvents and utilize these recycled ILs for reaction and process purposes.^15−17^

The physical properties of IL solvents (e.g., viscosity and hydrophilicity) are critical factors in choosing the appropriate recycling routes. The hydrophilicity, or miscibility with polar solvents, is largely determined by the molecular size of the IL anion, as smaller ions bear stronger solvation interactions than larger ones.^18^ Approaches to recycle IL solvents can be classified in two ways: (1) direct removal of impurities or target ILs or (2) combined processes. Distillation is a typical practice to separate single phase solution mixtures whose components possess differential boiling points. Researchers have reported the direct recycling of ILs by either making impurities or rarely ILs as distillates.^19−21^ Despite the operational simplicity, separation of compounds with high boiling points can be dramatically energy intensive,^22^ and it is unsuitable to remove nonvolatile impurities (e.g., inorganic ions) in the IL. Crystallization provides an alternative thermal phase-change approach to recover ILs from the solution containing nonvolatile impurities, although high energy inputs are still inevitable.^23,24^ Adsorption and extraction are two low energy consumption options for direct purification. Adsorption–desorption methods offer outstanding selectivity and scalability;^25,26^ nevertheless, the adsorbate-specific nature of the adsorption mechanism makes it laborious to find the proper adsorbents and it can be complicated when multiple contaminants must be removed. In direct extraction techniques, either impurity-rich or IL-rich phases can be the extracts, while aqueous solutions or organic solvents can be the washing agents.^27−29^ One prerequisite to employing such a method is that the recycled ILs are required to be immiscible with the washing agents for phase separation to be successful. Combined methods integrating different operations (e.g., evaporation, extraction, phase separation) enable tailored removal of multiple contaminants from a wide range of ILs.^30−32^

We previously reported a recyclability study on a matrix of six IL solvents used for a model Pt nanoparticle synthesis reaction, where an early stage techno-economic analysis was performed to guide the choice of IL solvents.^33^ The purification process was carried out in a 3D-printed, continuous-flow microfluidic recycler, in which the contaminated IL solvents were purified by biphasic liquid–liquid extraction and recovered by polymer membrane-based separation. Continuous-flow processes are often superior to legacy batch procedures that can be labor-intensive, poorly reproducible, scalable but with limited mass/heat transfer efficiency, environmentally hazardous, *etc*.^34^ Diverse mass transfer-enhanced designs for extraction and efficient mixing are supported in microfluidics.^35−38^ Meanwhile, liquid–liquid phase separation has been broadly studied and applied in this realm,^39−42^ where special attention has been paid to membrane separation that relies on differential wettability of a given membrane and Laplace pressure driving the separation of two immiscible liquids.^43^

Given the fact that most combined extraction and separation methods are based on multiphase liquid–liquid systems, examples of purifying IL solvents miscible with the washing agents are rare. One example can be found in our previous work where three IL solvents with complete water miscibility were recycled using supported IL membranes (ILMs),^33^ with water being a better washing agent than VOCs (i.e., hexanes) in this role.^17^ ILMs are polymeric membranes (e.g., polyvinyl fluoride) prewet by a hydrophobic IL that preferably permit the permeation of similar organic compounds while others are selectively excluded.^44^ In this case, the IL membrane is selective for the IL in the water-IL mixture. Although the reported recovery rates of the water-miscible ILs were observed to reach 70%, non-negligible amounts of polar-soluble impurities still remained and accumulated in the recycled IL products leading to degraded quality of Pt nanoparticles upon subsequent recycling and reuse.^33^ This is largely due to the inability of the ILM to retain organic impurities. It is nontrivial to solve a multicontaminant removal problem in IL recycling.

In this work, we describe a solvent recycling route for water-miscible ILs that consists of a step of extraction of contaminants followed by the recovery of target ILs. Here, water-miscible ILs with the triflate anion (OTf^–^) were the targets to be recycled. Hydrophobic ILs with bis(trifluoromethylsulfonyl)imide anion (NTf_2_^–^) were introduced as “accommodating agents”, and acidified water was the washing agent. This recycling route is abbreviated as “AAA” (accommodating agent-aided). The AAA strategy was implemented in a continuous-flow microfluidic process coupled with micromixing and membrane separation. A preliminary mixing and miscibility test among the three species (OTf^–^ IL, NTf_2_^–^ IL, and water) was conducted to study the pH-dependent partition behavior of OTf^–^ ILs between the NTf_2_^–^ IL and water phases. As proof of concept, several factors (e.g., IL recovery rates, and amounts of the residual impurities) were evaluated in the washing of 1-butyl-3-methylimidazolium triflate (BMIM-OTf) with water and 1-butyl-3-methylimidazolium bis(trifluoromethylsulfonyl)imide (BMIM-NTf_2_). Later, we performed recycling of an IL solvent, 1-butyl-1-methylpyrrolidinium triflate (BMPYRR-OTf), from the actual reaction mixture of a Pt nanoparticle synthesis, demonstrating that the application of the prototypical route was practical in a realistic chemical process.

## Results and Discussion

**Figure 1:**
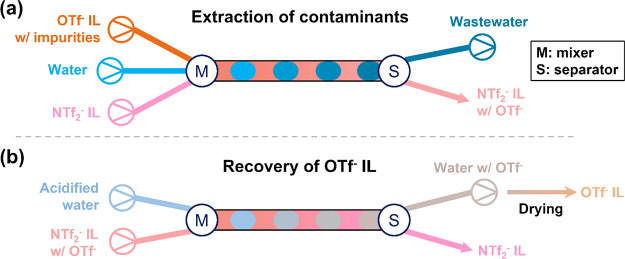
Schematics of the IL recycling process in continuous flow with two steps: (a) extraction of contaminants and (b) recovery of OTf^–^ ILs. Colors of the streams are based on the kinds of components involved and their according mixtures. In the biphasic flow, the aqueous phase is the dispersed phase represented by oval-like slugs, while the IL phase is the continuous phase.

The continuous-flow AAA IL purifying process contains two steps: (a) extraction of contaminants with water in the presence of NTf_2_^–^ (Figure 1a) and (b) recovery of OTf^–^ ILs from the NTf_2_^–^ accommodating agent using acidified water (Figure 1b). In the first step, a stream of hydrophilic (OTf^–^-based) IL to be recycled, a stream of hydrophobic (NTf_2_^–^-based) IL, and a stream of water are introduced into the flow process. Here, the NTf_2_^–^ IL phase and the aqueous phase form a biphasic slug flow in a micromixer with the OTf^–^ IL distributed at some partitioning ratio between the two phases. The pH of the water is selected based on the miscibility study described below to minimize the loss of OTf^–^ IL to the wastewater. The two phases are separated after extraction in a membrane separator. In the second step, acidified water at a relatively low pH value is used to strip the OTf^–^ IL from the washed IL product from the first step. The aqueous product carrying the target OTf^–^ IL from membrane separation is dried offline to remove water and complete the recovery step.

In designing the pH of water used in the AAA process, we performed a study of the partitioning behavior among water, OTf^–^ ILs (miscible with water), and NTf_2_^–^ ILs (immiscible with water). The miscibility study was carried out in batch. Equal amounts of OTf^–^ IL, NTf_2_^–^ IL, and water at various pH values were mixed and then phase separated via centrifugation. A second wash was performed by decanting the upper (water-rich) layer and adding fresh water to be in contact with the remaining lower IL phase followed by another cycle of mixing and separation. The retention factors of the OTf^–^ IL in the NTf_2_^–^ IL phase were calculated using eq 1. We assumed that the solubility of NTf_2_^–^ ILs in water is negligible.^45^
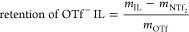
1where *m*_IL_ is the total mass of the IL phase, *m*_NTf2_ is the mass of NTf_2_^–^ IL added, and *m*_OTf_2__ is the mass of OTf^–^ IL added initially. A retention factor of 1 would indicate complete failure of the aqueous phase to separate the OTf^–^ IL from the NTf_2_^–^ IL; a retention factor of zero means that all OTf^–^ IL partitioned to the aqueous phase.

**Figure 2:**
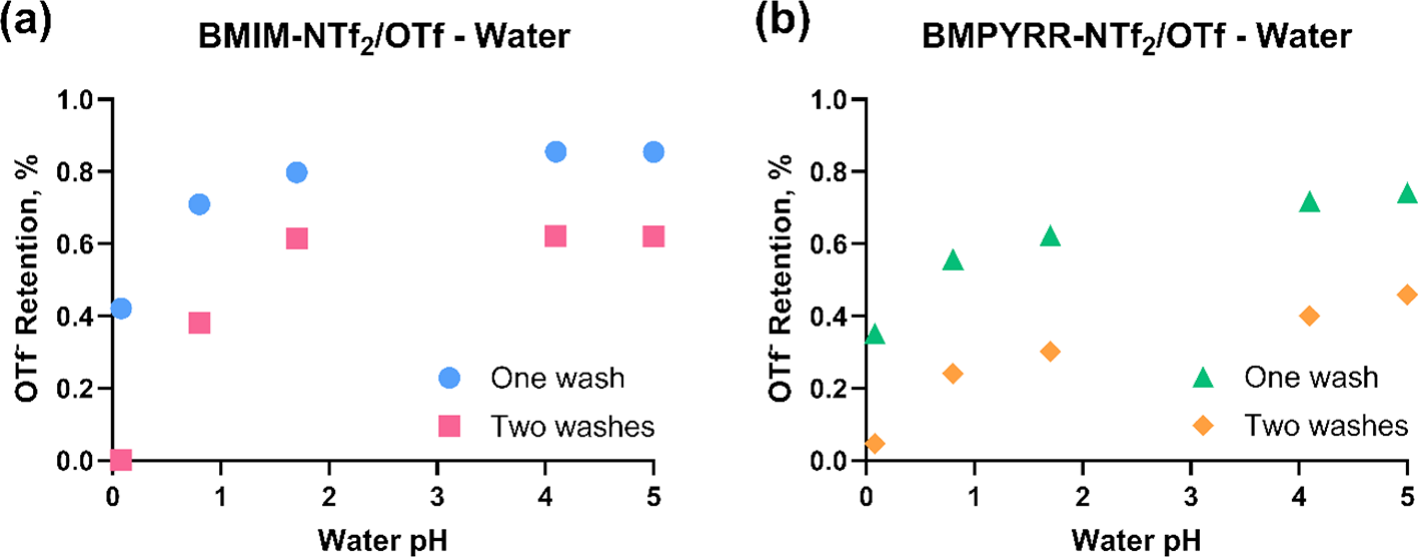
Retention factors of OTf^–^ ILs in the IL phase. (a) Mixture of BMIM-OTf, BMIM-NTf_2_, and acidified water at different pH values. (b) Mixture of BMPYRR-OTf, BMPYRR-NTf_2_, and acidified water at different pH values.

Figure 2 shows that for both IL-water systems, the retention factors of OTf^–^ ILs decrease with decreasing water pH, meaning that more of the OTf^–^ ILs partition to water at lower pH (higher partition coefficients, as shown in Figure S1). Retention factors of the OTf^–^ IL with BMPYRR^+^ as the cation are lower than those with BMIM^+^ as the cation at a given pH value. Note that the retention factor does not reach 100% even with DI water, which gives a pH value of ca. 5. Also, note that the second wash can remove more OTf^–^ IL from the IL phase, and the retention factors are nearly zero using two washes with water at pH = 0, indicating that no OTf^–^ IL is left in the NTf_2_^–^ IL. This pH-dependent distribution observation can guide the design of the aqueous phase used in the microfluidic implementation of IL recycling.

**Figure 3:**
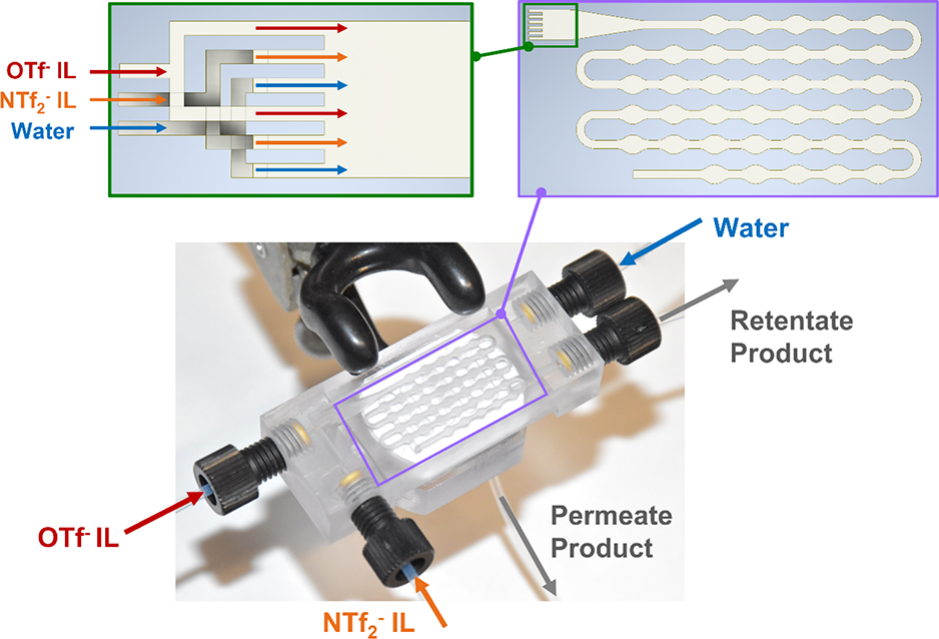
Picture of the 3D-printed recycler, specifying three input streams of OTf^–^ IL, NTf_2_^–^ IL, and water, and two output streams of the retentate and permeate phases after membrane separation. A diagram of the wavy channel for extraction is shown in the purple box. The six substreams after splitting of manifolds into the funnel-like merging mixer are depicted in the dark green box.

The two-step continuous-flow AAA process was executed in a 3D-printed microrecycler (Figure 3). After the infusion of the three streams, each of them was equally split into two branches in a manifold in the recycler. In a sequence as shown in Figure 3, all six substreams (channel cross-section = 200 μm × 200 μm) met in a funnel-like inlet that constricted from 2.2 to 0.6 mm. Here, a biphasic flow was established, with the aqueous phase dispersed into the NTf_2_^–^ IL phase and the OTf^–^ IL partitioning between the two. A video showing this flow pattern is available in the Supporting Information. A stable slug flow was formed at the end of the entry constriction. This flow entered a length of the channel with wavy walls and round turns designed to accelerate mass transfer and the extraction of contaminants. Diffusional mass transfer between the adjacent slugs was improved by the quick internal circulation and convective mixing within a given slug induced by the no-slip boundary condition.^42^ Mass transfer between and convective mixing within the slugs were intensified by the wavy walls that created an additional velocity profile in the direction perpendicular to the flow-forward direction, visualized by the “expansion” of the slug in the widening area and the “shrinkage” of the slug in the narrowing area.^33,46^ Round turns were also designed to trigger Dean flow, which harnessed a secondary flow field inside the liquid slug to promote the recirculation of the fluid, countering otherwise slow mixing in the stagnant layer near the channel walls.^47^ After extraction, the slug flow passed through a phase separation section where a hydrophobic PTFE membrane (pore size = 0.1 μm) was employed. The separated phases were collected for the next use. Note that in the step of OTf^–^ IL recovery, streams of OTf^–^ IL and NTf_2_^–^ IL were both replaced with the streams of mixed IL product from the first step. This system is modular, such that the numbers of stages in each step can be modified according to need. For example, a two-stage impurity extraction step means the washed IL product will be fed back to the recycler inlet for a second contact with the fresh washing agent. Three infusion flow rates and the withdrawal flow rates that together controlled the membrane separation efficiency were determined in preliminary tests for the system to achieve perfect separation of the two phases.

We initially performed a study in the absence of any impurities to characterize the ability of this system to recover an OTf^–^ IL and reuse an accommodating IL. We used as-received BMIM-OTf as the OTf^–^ IL source and as-received BMIM-NTf_2_ as the accommodating agent. DI water and acidified water at pH = 0 were used in the first and second steps, respectively. We used one stage for the first step (impurity extraction) and two for the second step (IL recovery). To demonstrate the reusability of BMIM-NTf_2_ as an accommodating agent, BMIM-NTf_2_ from a given run was used directly in the next round of recycling (i.e., the NTf_2_^–^ IL input in the step of impurity extraction). Table 1 summarizes the recovery rates of OTf^–^ and NTf_2_^–^ ILs and amounts of OTf^–^ IL in the NTf_2_^–^ IL product, and *vice versa*.

**Table 1:** Results of the Contaminant-Free Separation Study^a^

	**BMIM-OTf recovery rate (v/v, %)**	**BMIM-NTf_2_ recovery rate (v/v, %)**	**BMIM-NTf_2_ in BMIM-OTf product**(mol %)	**BMIM-OTf in BMIM-NTf_2_ product**(mol %)	**IL in wastewater (wt %)**
1× process	83.9	90.9	13.5	0	10.8
2× process	83.0	91.5	13.0	0	9.0
3× process	86.5	92.5	11.5	0	10.7

a Recovery rates are presented in volumetric ratios. IL content in the other IL is calculated in mole ratios. IL content in the wastewater is measured in weight percentages. 1× process refers to using as-received BMIM-NTf_2_, while 2× and 3× refer to the times of reuse of BMIM-NTf_2_ from the previous process.

The recovery rates of both the target BMIM-OTf and the accommodating agent BMIM-NTf_2_ were 83–93% by volume and were stable over 3× reuses. The IL content in the other IL product was calculated based on the integration of peaks in the ^19^F NMR spectra (Figure S2c). It is noted that ca. 12% of BMIM-NTf_2_ was detected in the recycled BMIM-OTf products that were retrieved from the aqueous phase in the recovery step. In the miscibility study, we first hypothesized that the NTf_2_^–^ ion itself is highly immiscible in the aqueous phase. Here, the nonzero content of BMIM-NTf_2_ in BMIM-OTf products *de facto* disclosed a nonzero dissolution of the NTf_2_^–^ ILs in the aqueous phase, which to some extent agreed with the results reported previously that the solubility of an IL in the aqueous phase could change at different concentrations of another hydrophilic IL or salts doped in the mixture.^48,49^ In contrast, no detectable OTf^–^ ions were observed in BMIM-NTf_2_, as illustrated by the clean single resonance in the ^19^F NMR spectrum (Figure S2d), reflecting that the selected water acidity was capable of capturing all BMIM-OTf from the BMIM-NTf_2_ phase after two recovery stages. The non-100% recovery rates of both ILs indicated a loss of ILs. ILs could be lost during device operation (e.g., in the dead volume of the channel and recycler) and sample handling. Inevitable dissolution of the OTf^–^ ILs in the wastewater was another source of losing ILs, as shown by the IL content in wastewater quantified in Table 1 and as indicated by the observation in the miscibility study that ca. 20% of the OTf^–^ IL would stay in the aqueous phase even with DI water (high pH). The recovery rates of ILs can be expected to further increase by additional treatments, for example, an extra step to recover the IL from the wastewater.

**Figure 4:**
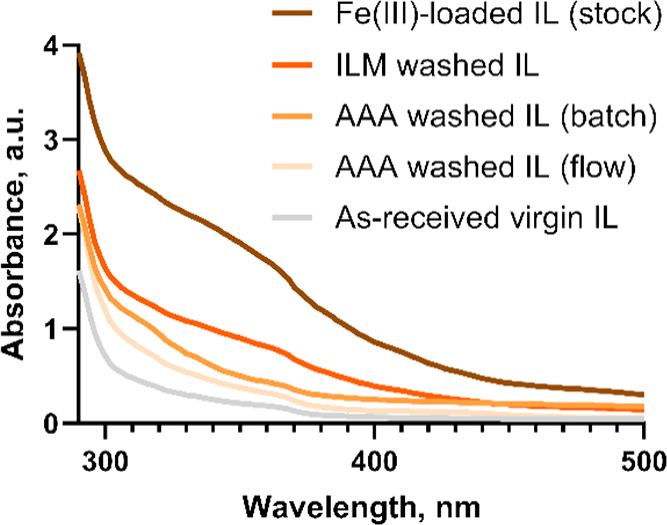
UV–vis absorbance spectra of varying BMIM-OTf samples.

To study the efficiency of extracting impurities from the OTf^–^ IL with the recycler, Fe(NO_3_)_3_ was added to the as-received BMIM-OTf solvent. Fe(III) ions are a good indicator of the water-IL biphasic extraction because they are spectrophotometrically detectable at 310 nm and complete mass transfer from the IL phase to the aqueous phase can occur.^50^ Fe(III) ion-loaded BMIM-OTf, as-received BMIM-NTf_2_, and DI water were injected into the recycler for the step of extraction of Fe(III) ions, and acidified water at pH = 0 was used to recover the washed BMIM-OTf from the IL phase in two stages for the second step. Interestingly, during membrane separation, the aqueous phase became the permeate phase, passing through the normally hydrophobic PTFE membrane. This is in contrast to what was observed in the preliminary study with no inorganic contaminants in which the hydrophobic IL phase acted as the permeate. This inversion of the retentate and permeate could stem from the introduction of Fe(III) ions; a rust-brown color could be seen on the membrane, indicating that Fe(III) ions were adsorbing on the membrane and altering the wettability. As a result, the retentate IL phase was collected in the first step, instead of the permeate. To evaluate the performance of the flow recycling, we compared it with standard batch recycling. In the batch procedure, the same reagents at the same volumetric ratios were mixed and then phase separated via centrifugation. We also benchmarked this system performance against an ILM separator, as described above and deployed in our previous work.^33,50^ This separator utilized an identical 0.1-μm-pore PTFE membrane incubated with BMIM-NTf_2_ overnight prior to use. The retentate phase was collected as the washed IL product, in line with the operation of the two-step flow process. Metrics to compare the three methods are given in Table 2 and Figure 4.

**Table 2:** Summary of Recycling Performance with Different Methods^a^

	**ILM**	**AAA in batch**	**AAA in flow**
BMIM-OTf Recovery Rate (v/v, %)	50.5	87.0	78.6
BMIM-NTf_2_ Recovery Rate (v/v, %)	N.A.	90.8	87.3
Water Conc. in Re. BMIM-NTf_2_ (wt%)	N.A.	4.9	2.2

a Recovery rates are presented in volumetric ratios. Water concentrations in recycled BMIM-NTf_2_ are presented in weight percentages. Recovery rate and water concentration are unavailable in the ILM method due to process difference.

ILM had the lowest recovery rate of BMIM-OTf of the three approaches (Table 2). This makes it clear that the separation of the homogeneous mixture merely relying on intermolecular interactions between the prewet hydrophobic IL and the target hydrophilic IL is of limited utility. Also, the residence time possible in flow is likely insufficient for equilibrium ILM separations, as literature reports of such approaches describe incubation times up to 50 h in batch.^44^ Recovery rates using the AAA batch and flow processes slightly varied due in great part to the material loss in the flow device, as discussed above. The recovery rate deficit can be expected to be alleviated or even inverted when scaling up, since the dead volume of the flow system is constant and the fraction of liquid lost to this dead volume will approach zero as reagents are continuously processed. Moreover, the flow process produces a BMIM-NTf_2_ phase with less water present at the end of the process (2.2% versus 4.9%), resulting from the hydrophobic membrane used in the recycler also assisting in filtering water content. Figure 4 illustrates that all three methods managed to remove Fe(III) ions to various degrees. The washed IL from the ILM method still carried the highest concentration of Fe(III) among the three, while the Fe(III) concentration level closest to the as-received virgin IL was found in the IL product purified by the AAA route in flow, reflecting not only the extraction but also the adsorption of Fe(III) on the membrane.

**Figure 5:**
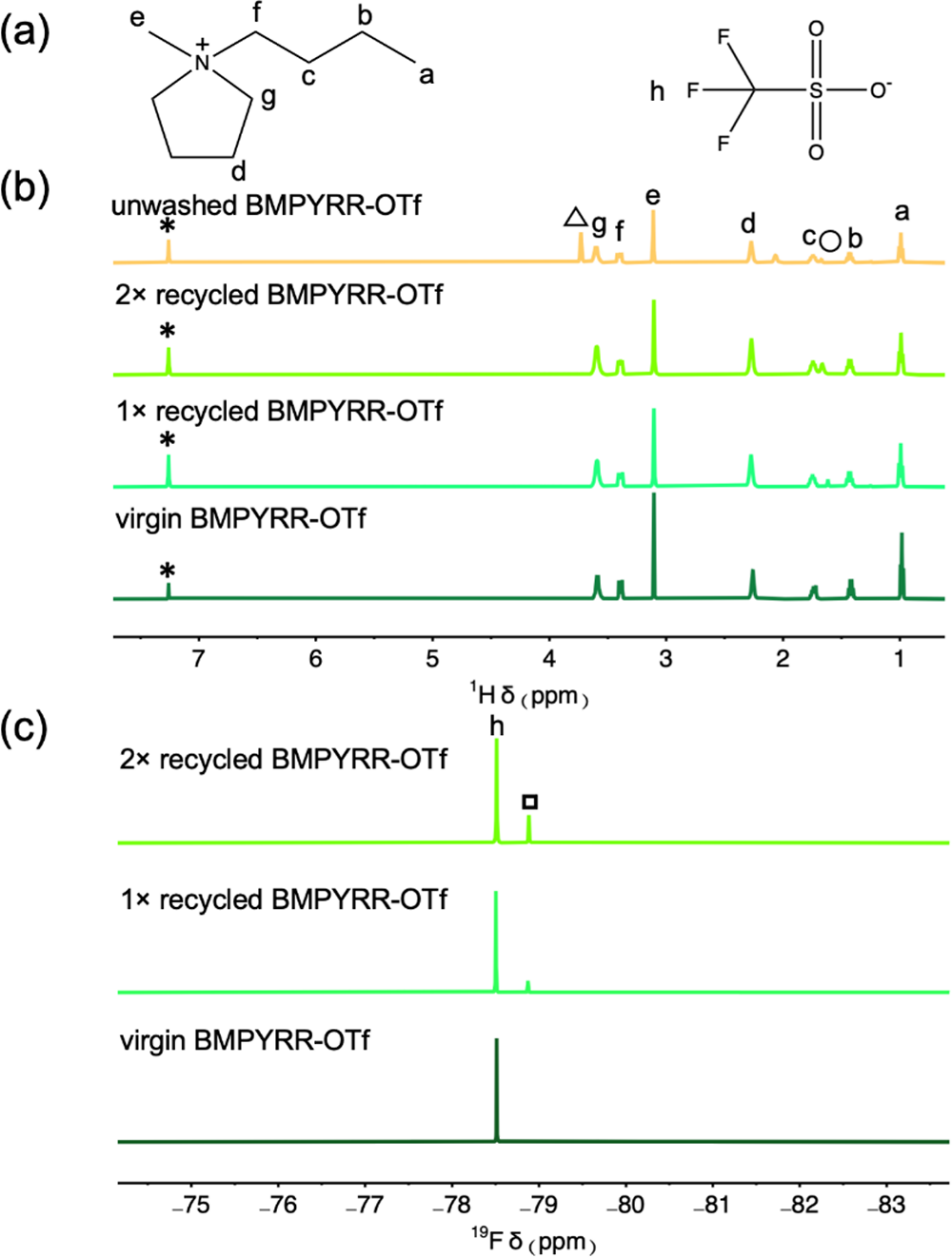
(a) Structure of the BMPYRR^+^ cation (left) and OTf^–^ anion (right). (b) ^1^H NMR spectra of virgin, 1× recycled, 2× recycled BMPYRR-OTf, and unwashed BMPYRR-OTf. (c) ^19^F NMR spectra of virgin, 1× recycled, and 2× recycled BMPYRR-OTf. The empty circle (o) denotes water at 1.56 ppm. The open triangle denotes ethylene glycol. The open square denotes the ^19^F resonance from the NTf_2_^–^ anion. Asterisks represent the residual nondeuterated solvent peak of chloroform.

Finally, we applied this water-miscible IL recycling system to a realistic chemical process scenario, that is, the purification of the used IL solvent (BMPYRR-OTf) in a polyol reduction of Pt(II) to synthesize colloidal Pt nanoparticles.^17^ The postreaction mixture after separation of the Pt nanoparticles contained large amounts of impurities: ethylene glycol (unreacted reducing agent), PVP (excess caping agent), K_2_PtCl_4_ (unreacted metal salt), some nonisolable nanoparticles, and other reaction byproducts. Here, water is a green washing agent to extract waste from the IL solvent, and recent research unveiled that the removal of Pt can be realized by using acidified water (low pH).^51^ The post-reaction mixture was passed through the recycler along with as-received BMPYRR-NTf_2_ and acidified water at pH = 2. The selection of water pH here was based on balancing the recovery rate of the IL and the efficient removal of Pt^2+^ impurities. Using water at high pH (e.g., DI water) can salvage most of the OTf^–^ IL at the cost of Pt extraction efficiency, while using water at low pH (e.g., pH = 0) can maximize the extraction performance at the expense of losing more target IL. In this work, we chose to balance these effects by choosing an intermediate pH. The mixed IL product containing BMPYRR-NTf_2_ and BMPYRR-OTf was transferred to the recovery step to obtain recycled BMPYRR-OTf. After a two-stage recovery, 1× recycled BMPYRR-OTf was analyzed and reused for another Pt nanoparticle synthesis reaction. After the reaction, the used IL solvent was washed again to yield 2× recycled IL. Figure 5 compares the solution ^1^H and ^19^F NMR spectra of the as-received virgin IL, 1× , 2× recycled, and unwashed ILs (post-reaction solvent). This demonstrates that the recycled IL solvent is chemically unchanged after the first and second recycling, with no detectable presence of reaction byproducts or other impurities, compared to the unwashed IL solvent that contains a significant amount of ethylene glycol. As reference, the post-reaction IL solvent was also purified using the previous ILM method,^33^ and Figure S3 shows a significant amount of ethylene glycol still persisted in the IL recycled. The mole fractions of BMPYRR-NTf_2_ in the 1× and 2× recycled BMPYRR-OTf products were estimated to be 4.2 and 7%, respectively, based on the integration of the peaks in the ^19^F NMR spectra (Figure 5c), which display the different chemical shifts for the OTf^–^ and NTf_2_^–^ anions. Inorganic impurities of Pt salts and nonisolable Pt nanoparticles were monitored by ICP-OES, and the result reveals that 861 and 518 ppm of Pt (limit of detection = 9.7 ppm) were found in the IL samples before and after the wash. Recovery rates of the BMPYRR-NTf_2_ accommodating agent were 87.8 and 93.6% for the first and second recycle, while the BMPYRR-OTf solvent recoveries were 50.9 and 48.6%, correspondingly. It is unsurprising that the recovery of OTf^–^ solvents only reached moderate numbers, in that ca. 40% of the BMPRYY-OTf was likely to be distributed in the acidified water at pH = 2 (Figure 2b). The choice of pH is an engineering parameter that can be tuned to favor extraction efficiency or IL recovery; a pH of 2 represents a compromise between these objectives. Accumulation of black particulate matter (likely residual Pt particles) over time on the membrane was observed in the extraction step; however, degradation of the separation efficiency of the membrane was not observed during the entire process. Note that the separation efficiency could be potentially impacted by the ongoing particle accumulation, which may require remodification of the flow rates for perfect separation or replacement of the membrane.

## Conclusions

We report a route to recycle ionic liquid solvents miscible with water or other polar solvents by pairing biphasic liquid–liquid extraction with membrane-based separation. Solvent recovery was enabled by taking advantage of the pH-dependent partition coefficients of the target hydrophilic IL between the aqueous washing phase and the hydrophobic IL phase. This recycling route was executed in a continuous microfluidic process using a 3D-printed recycler prototype. Recovery rates >80% were observed for the hydrophilic BMIM-OTf IL in the presence of BMIM-NTf_2_ and water. Furthermore, purification of metal-ion-contaminated IL solvents in the flow process outperformed the analogous batch procedure as well as a previously reported method of IL membrane separation. The accommodating agent-aided route was further applied to a realistic case in which the recycling of IL solvents from a Pt nanoparticle synthesis was demonstrated. NMR spectra and ICP-OES data showed a successful removal of organic byproducts and impurities and a 40% stripping of inorganic Pt residues from the reaction, in a one-stage-only extraction step over two cycles. This work demonstrates several engineering parameters that can be tuned to optimize recovery of water-miscible ILs in a water-based impurity extraction process: (1) using less acidified water, or even DI water, to extract impurities coupled to more stages of extraction; (2) adding an additional step to recover target ILs from the wastewater; and (3) lowering the process temperature such that the water-miscible IL tends to partition more easily to the IL phase. Process engineers must balance the removal efficiency of impurities, the sustainability gains of using recycled ILs, and the operational costs associated with the additional process steps. Our previous early stage techno-economic analysis demonstrated that among the IL solvents evaluated, the water-miscible BMPYRR-OTf solvent led to a lowest cost of the Pt-based nanoparticle catalyst produced, but the quality of nanoparticles was heavily compromised because of insufficient removal of impurities during recycling.^33^ This work shows that the recycling of water-miscible ILs is feasible and provides an industrially realistic route to recycle these ILs.

Further, this recycling process is expected to be scaled up via parallelization.^52^ A 56 parallel line system would be capable of purifying the IL solvent from a 100 mL reaction per hour, with minimal labor cost due to the automatic, continuous operation. In comparison, traditional by-hand extraction and separation using separation funnels can purify the same amount of solvent within 1 h; however, this requires intensive human operation that would significantly increase the total cost. In scaling up the process to meet industrial requirements, the final drying process can be implemented in continuous-flow evaporators.^53^

## Experimental Procedures

### Materials

1-Butyl-3-methylimidazolium bis(trifluoromethylsulfonyl)imide (BMIM-NTf_2_, 99%) and 1-butyl-1-methylpyrrolidinium triflate (BMPYRR-OTf, 99%) were purchased from IoLiTec and used as received. 1-Butyl-3-methylimidazolium trifluoromethanesulfonate (BMIM-OTf, 97%), K_2_PtCl_4_ (99.9%), polyvinylpyrrolidone (PVP, MW = 55,000), ethylene glycol (99.8%), and Fe(NO_3_)_3_•9H_2_O (98%) were all purchased from Sigma-Aldrich and used as received. 1-Butyl-1-methylpyrrolidinium bis(trifluoromethanesulfonyl)imide (BMPYRR-NTf_2_, 98%) was purchased from TCI Chemicals and used as received. Acidified water at different pH levels was prepared by diluting a nitric acid solution (Supelco, 68–70%) with deionized (DI) water. The pH values were verified by a pH meter (Jenway 3510).

### Miscibility Study on NTf_2_^–^ IL, OTf^–^ IL, and Water Mixture

In the pH effect study, equal volumes of BMIM-NTf_2_, BMIM-OTf, and acidified water (100 μL, each) were added to a 1.5 mL conical tube. The mass of all three components and the empty tube was measured. The mixture was vortex mixed for 1 min and then phase separated *via* centrifugation (1 min, 2,500 rcf). The upper aqueous layer was decanted from the tube, and the lower IL layer was dried at 70 °C for 2 h prior to the mass measurement. For a second wash, 100 μL of fresh acidified water was added to the tube again, followed by the same mixing, separation, and weighing procedures. Miscibility tests using BMPYRR-NTf_2_ and BMPYRR-OTf were carried out with the same procedures. In the OTf^–^ content study, 100 μL of BMIM-NTf_2_ and acidified water at pH = 1, respectively, and BMIM-OTf of varying volumes were added to a 1.5 mL conical tube, followed by the mixing, separation, and weighing procedures.

### Fabrication of 3D-Printed Microrecycler

Designs of the microfluidic recycler device were finished in Autodesk Inventor Professional 2022 (details and measurements are available in the Supporting Information). The device was fabricated by a stereolithographic 3D printer (Asiga, MAX X UV385) with a transparent methacrylate-based resin, GR-10 (Pro3dure Medical). The as-printed device was thoroughly washed in three consecutive 2-propanol (IPA) baths right after being removed from the printer. IPA was also injected using a 20 mL syringe to flush the internal channel. The device was then immersed in IPA in a glass beaker and placed in an ultrasonic bath (Elma, E15H) at 30 °C for 20 min and air-dried completely prior to the next step. A piece of polytetrafluoroethylene (PTFE) membrane with pore size 0.1 μm (Sterlitech) was cut to a proper shape and placed on the separation position of the recycler. A quick-cure epoxy (Bob Smith Industries) was applied to combine the two halves of the recycler. The recycler sat for 2 h to allow full curing of the epoxy. 200 μL of either BMIM-NTf_2_ or BMPYRR-NTf_2_ was injected into the recycler to prewet the membrane, and the excessive IL was withdrawn after the 2 h incubation.

### Mixing and Separation Study in Continuous Flow

As-received BMIM-NTf_2_, BMIM-OTf, and DI water were loaded in 10 mL of Luer-lok plastic syringes (BD). Three syringe pumps (Harvard Apparatus, 11 Plus) were operated in infusion mode to feed the three streams into the recycler, and a syringe pump (Chemyx, Fusion 200) was operated in withdrawal mode to collect the retentate product coming from the upper outlet of the recycler. Flow rates of all streams were executed as follows: 60 μL/min for BMIM-NTf_2_ infusion, 30 μL/min for BMIM-OTf infusion, 100 μL/min for DI water infusion, and 105 μL/min for retentate withdrawal. The permeate product coming from the lower outlet was collected by a 15 mL tube. Connection PTFE tubing (1.6 mm OD × 0.8 mm ID) and parts in the flow process were purchased from Cole-Parmer and IDEX Health & Science, respectively. The permeate IL product collected and acidified water (pH = 0) were transferred to new syringes and reloaded to two syringe pumps, respectively. In the OTf^–^ IL stripping step, the IL and acidified water were infused into a new recycler in which the IL stream was split into two parallel streams by a T-shaped manifold before entering the recycler. A withdrawal syringe pump was also set to the upper outlet of the recycler, while permeate product from the lower outlet was collected by a tube. Flow rates of IL infusion (before splitting), acidified water infusion, and retentate withdrawal were 60, 150, and 165 μL/min, respectively. The permeate product collected was reloaded to a syringe for a second stripping process with same setup; however, the withdrawal flow rate was modified to 152 μL/min. To calculate the BMIM-NTf_2_ recovery rate, the volume of the permeate product collected from the second wash was measured with a graduated cylinder. To calculate the BMIM-OTf recovery rate, retentate products from both two stripping steps were joined and transferred to a glass beaker and placed in an oven at 70 °C to remove water. Drying was considered complete when the mass of the liquid remained constant for 30 min. The volume of the dried product was then measured by a graduated cylinder. The IL content in the other IL product was calculated through the integration of peaks in the ^19^F NMR spectra that report on the mole fractions of the fluorine atoms in the two anions.

### In-Batch Purification of Fe(III)-Loaded BMIM-OTf in the AAA Route

In a standard procedure, a 1.93 mg/mL solution of Fe(NO_3_)_3_ in BMIM-OTf was prepared by thoroughly dissolving the salt in the IL in an ultrasonic bath. 600 μL of as-received BMIM-NTf_2_, 300 μL of Fe(III)-loaded BMIM-OTf, and 300 μL of DI water were added to a 5 mL centrifuge tube. Upon vortex mixing for 2 min, the tube was centrifuged (1 min, 2,500 rcf) to result in clear phase separation. The supernatant was removed, and 2,000 μL of acidified water (pH = 0) was added. The liquid mixture was vortex mixed for 2 min and phase separated through centrifugation (1 min, 2,500 rcf). The upper layer was transferred to a 30 mL glass beaker, and 2000 μL of fresh acidified water (pH = 0) was then added to the tube for a second wash with the same mixing and separation procedure. The upper layer was also transferred to the beaker which was later placed in the oven for drying.

### In-Flow Purification of Fe(III)-Loaded BMIM-OTf in the AAA Route

Three infusion pumps (as-received BMIM-NTf_2_, Fe(III)-loaded BMIM-OTf from the stock solution prepared above, and DI water) and a withdrawal pump were set up using flow rates of 60, 30, and 90 μL/min, respectively. The continuous-flow process followed the as-mentioned procedures of the mixing and separation study. The retentate IL product in the withdrawal syringe was prepared for two consecutive BMIM-OTf stripping steps (procedures referred to below), where flow rates were entered as IL infusion in 60 μL/min, acidified water infusion in 150 μL/min and withdrawal in 165 μL/min (first step), and 152 μL/min (second step). The retentate products from two steps were collected and dried completely in an oven prior to analysis.

### In-Flow Purification of Fe(III)-Loaded BMIM-OTf in ILM Separation

A membrane separator was designed as previously reported^50^ and fabricated using the above-described 3D-printing technique. The separator was prewet thoroughly with BMIM-NTf_2_. Fe(III)-loaded BMIM-OTf from the stock solution and DI water were premixed at the volumetric ratio of 1:1 in batch. The single phase mixture was fed into the separator in 100 μL/min and the retentate phase was withdrawn in 50 μL/min. After separation, the permeate phase was put in the oven to remove all of the water.

### Absorbance Spectrophotometry

80 μL aliquots of the samples were added by a pipettor to a 96-well plate (Celltreat Scientific Products, nontreated). Spectral scanning from 290 to 500 nm was conducted by a microplate reader (BioTex, Synergy H1), where steps of 2 nm in normal speed at 20.8 °C were set.

### Synthesis of Pt Nanoparticles

In a standard procedure, 42.1 mg (0.100 mmol) of K_2_PtCl_4_ was dissolved in 2.7 mL of ethylene glycol. Separately, 227.2 mg of PVP was added to 8.0 mL of the BMPYRR-OTf in a two-neck round-bottom flask equipped with a condenser and septum. The PVP was dissolved in BMPYRR-OTf by heating it in a thermostatically controlled oil bath at 150 °C for 10 min, giving a clear solution. The solution of K_2_PtCl_4_ in ethylene glycol was then hot injected into the BMPYRR-OTf and PVP solution and maintained at 150 °C for 30 min. The solution was thermally quenched in an ice bath. The reaction mixture was transferred to a 50 mL centrifuge tube, and 30 mL of acetone was added to precipitate the Pt nanoparticles. The supernatant containing the BMPYRR-OTf was saved, and the acetone and other volatiles were removed *in vacuo*. The resulting solution was further purified with acidified water using a continuous flow recycler.

### In-Flow Purification of BMPYRR-OTf from Pt Nanoparticle Synthesis

The post-reaction mixture from Pt nanoparticle separation, as-received BMPYRR-NTf_2_, and acidified water (pH = 2) were injected into the recycler by three syringe pumps in 30, 60, and 100 μL/min, respectively. A corresponding syringe pump running simultaneously at 110 μL/min was used to withdraw the retentate. The resulting IL permeate was taken to perform a two-time BMPYRR-OTf stripping process in which infusion of IL stream and acidified water (pH = 0) stream for both steps followed 80 and 130 μL/min, respectively. The withdrawal flow rates were modified from 135 (the first step) to 130 μL/min (the second step). Flow process parameters and drying details followed the procedures mentioned above.

### Nuclear magnetic resonance (NMR) Spectroscopy

NMR spectra (^1^H and ^19^F) were collected on a Varian 500 MHz VNMRS spectrometer with 16 scans. CDCl_3_ was used as the deuterated solvent. The concentration of each sample in the NMR tube was kept constant with the addition of 5 μL of the sample into 800 μL of CDCl_3_.

## Supporting Information Available

Additional partition coefficient plots, NMR spectra, and drawings of the 3D-printed device (PDF)
Inlet flow pattern (MOV)

## Supplementary Material

ie3c03312_si_001.pdf

ie3c03312_si_002.mov
