# Supplementary material for: A Multistep, Multicomponent Extraction and Separation Microfluidic Route to Recycle Water-Miscible Ionic Liquid Solvents
Source: Ind Eng Chem Res. 2023 Dec 20;63(1):489–97. doi: 10.1021/acs.iecr.3c03312 (PMC10785803; doi:10.1021/acs.iecr.3c03312)
Supplement: Supplementary file 1 — ie3c03312_si_001.pdf [file ie3c03312_si_001.pdf]

Supporting Information for

A Multistep, Multicomponent Extraction and Separation Microfluidic Route to Recycle Water-Miscible Ionic Liquid Solvents

Bin Pan,[a] Lanja R. Karadaghi,[b] Richard L. Brutchey,*[b] and Noah Malmstadt*[abcd]

[a] *Mork Family Department of Chemical Engineering and Materials Science, University of Southern California, 925 Bloom Walk, Los Angeles, California 90089-1211, United States*
[b] *Department of Chemistry, University of Southern California, 840 Downey Way, Los Angeles, California 90089-0744, United States*
[c] *Department of Biomedical Engineering, University of Southern California, 1042 Downey Way, Los Angeles, California 90089-0260, United States*
[d] *USC Norris Comprehensive Cancer Center, University of Southern California, 1441 Eastlake Ave, Los Angeles, California 90033, United States*

E-mail: brutchey@usc.edu
E-mail: malmstad@usc.edu

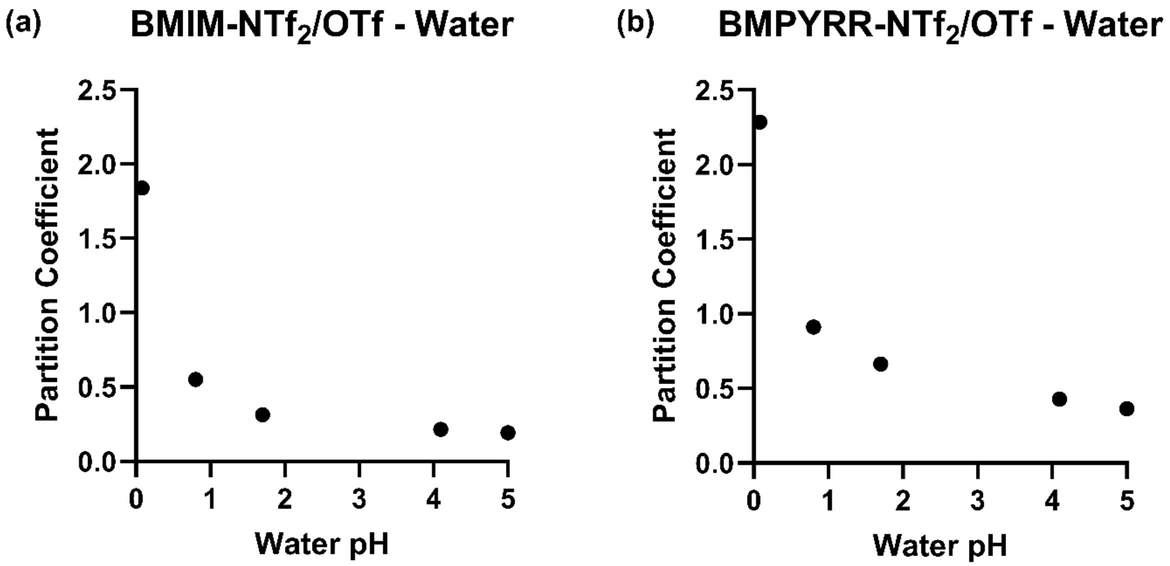

Figure S1. Partition coefficients for two water-IL systems. (a) Three-component mixture of BMIM-NTf_2, BMIM-OTf, and acidified water at different pH values. (b) Three-component mixture of BMPYRR-NTf_2, BMPYRR-OTf, and acidified water at different pH values. Partition coefficients were calculated based on the equation below.

$$Partition\ Coefficients = \frac{Mass\ of\ OTf^{-}\ IL\ in\ Aqueous\ Phase}{Mass\ of\ OTf^{-}\ IL\ in\ NTf_2^{-}\ IL\ Phase}$$

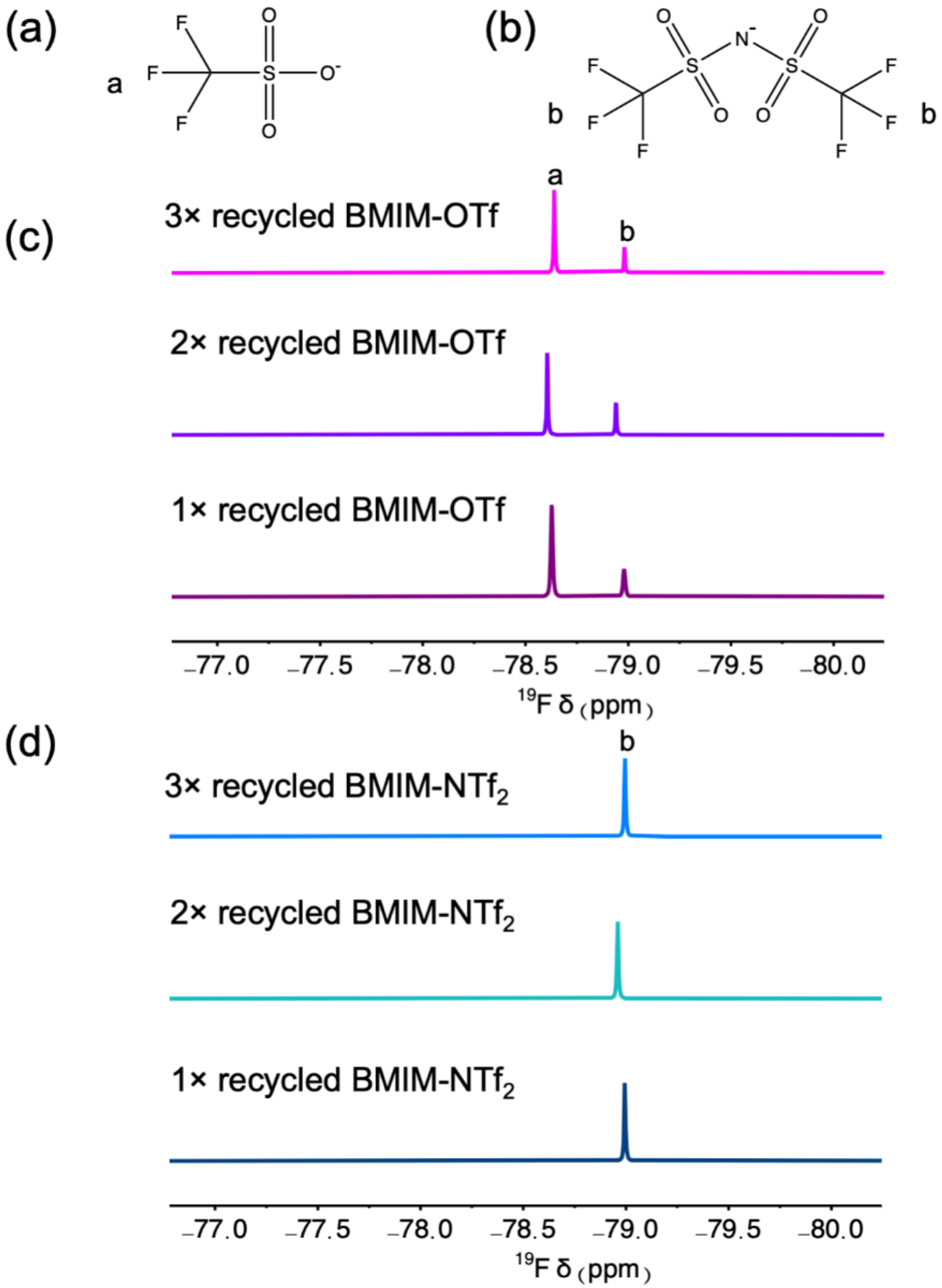

Figure S2. Structures of (a) OTf^- anion and (b) NTf_2^- anion. (c) ^{19}F NMR spectra of 1× recycled, 2× recycled, and 3× recycled BMIM-OTf. (d) ^{19}F NMR spectra of 1× recycled, 2× recycled, and 3× recycled BMIM-NTf_2.

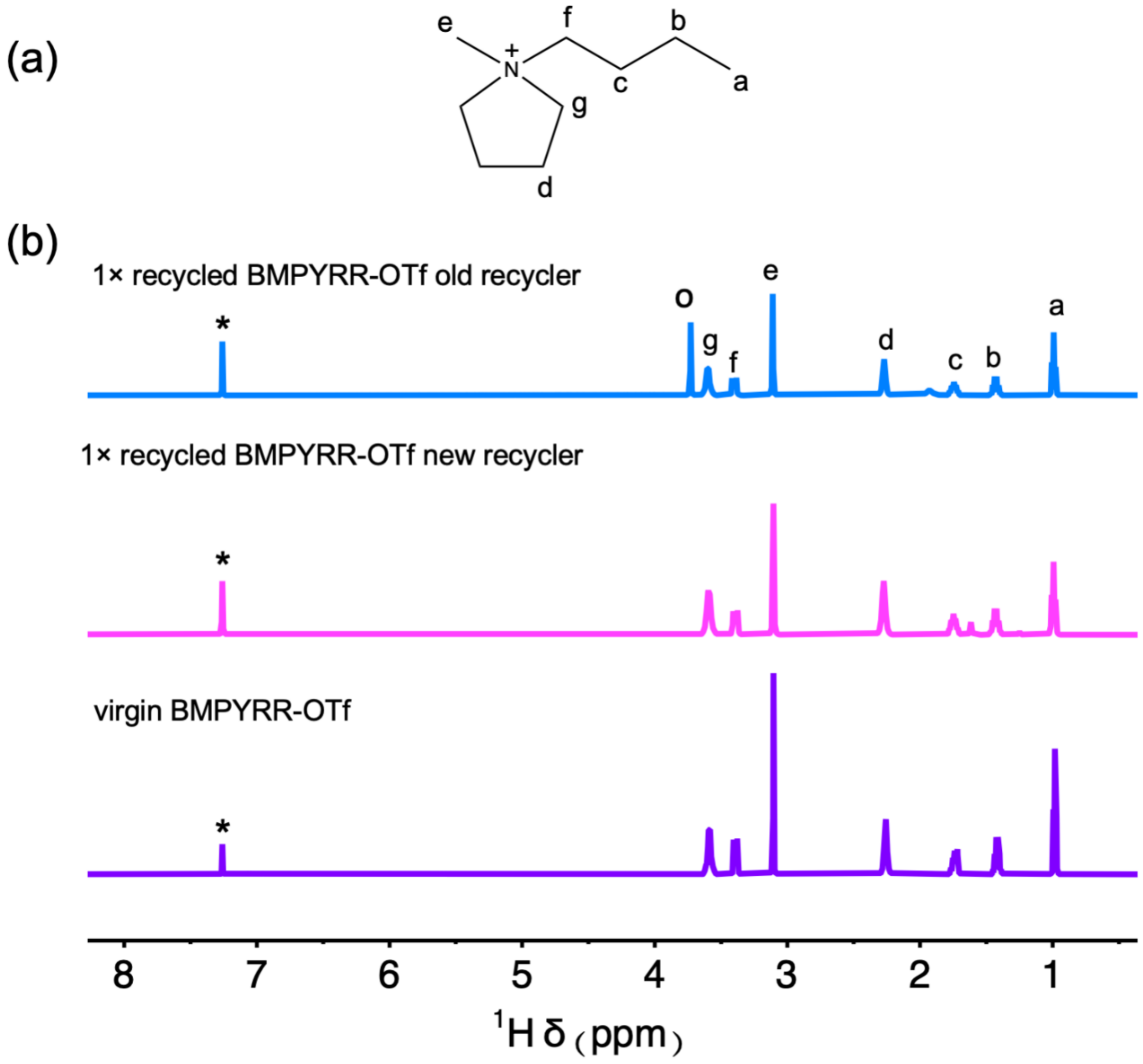

Figure S3. (a) Structure of $BMPYRR^+$ cation. (b) ^1H NMR spectra of virgin BMPYRR-OTf, 1× recycled using the new recycler, and 1× recycled using the old recycler. The "o" represents ethylene glycol present in the NMR spectra using the old recycling method. New recycler refers to the recycler using the AAA route in this work. Old recycler refers to the recycler used in our previous work.[1]

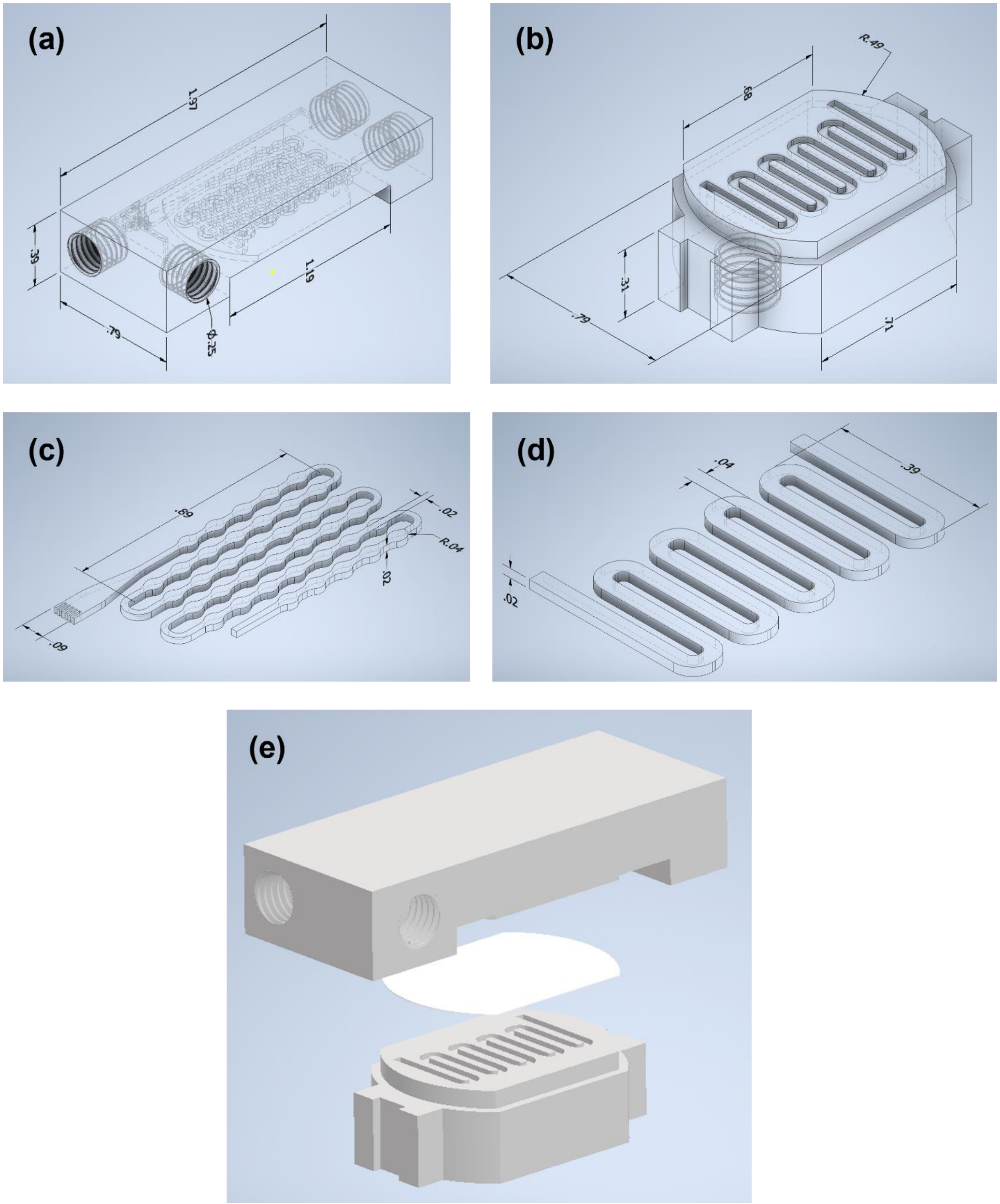

Figure S4. Drawings and details of the 3D-printed recycler. All measurements are in the unit of inches. (a) Upper part of the recycler. (b) Lower part of the recycler. (c) Shape of the channel of extraction section. (d) Shape of the channel of separation section. (d) Assembly view of the upper part, lower part and the membrane used (in white).

(a) (b)

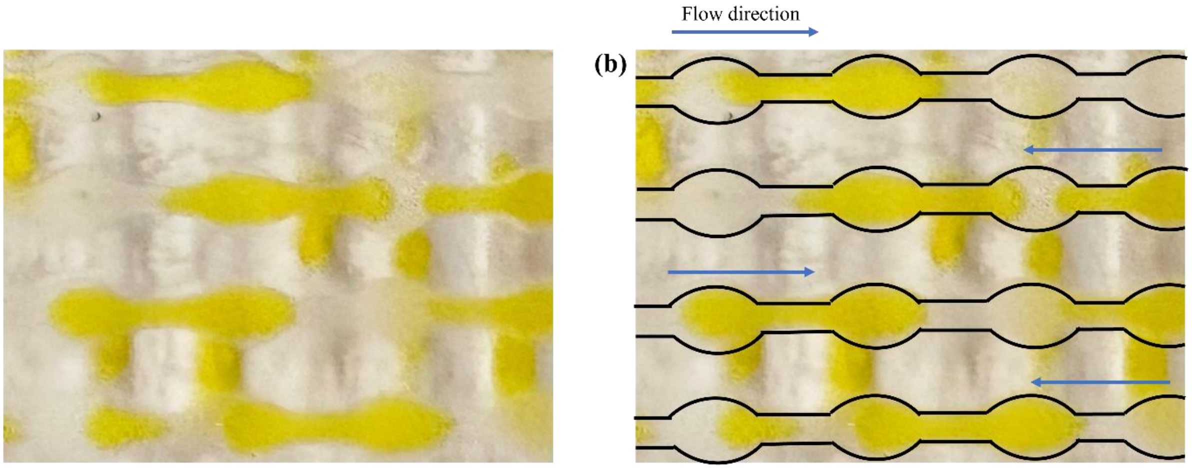

Figure S5. (a) Flowing slugs can be seen in the horizontal extraction channels in the recycler. Yellow liquid slugs represent dyed aqueous phase; transparent slugs represent IL phase. (b) Channel outlines are traced in black. Note: some yellow dye is visible in separation channels in the layer below.

Reference

1. Karadaghi, L. R.; Pan, B.; Malmstadt, N.; Brutchey, R. L. A techno-economic approach to guide the selection of flow recyclable ionic liquids for nanoparticle synthesis. *RSC Sustainability* 2023, Article ASAP. https://doi.org/10.1039/D3SU00182B
